# Differential diagnosis of demyelinating diseases: what's new?

**DOI:** 10.1590/0004-282X-ANP-2022-S109

**Published:** 2022-08-12

**Authors:** Ana Beatriz Ayroza Galvão Ribeiro Gomes, Tarso Adoni

**Affiliations:** 1 Universidade de São Paulo, Faculdade de Medicina, Hospital das Clínicas, Departamento de Neurologia, São Paulo SP, Brazil.; 2 Hospital Sírio-Libanês, Centro de Esclerose Múltipla, São Paulo SP, Brazil.

**Keywords:** Multiple Sclerosis, Neuromyelitis Optica, Myelin-Oligodendrocyte Glycoprotein, Diagnosis, Esclerose Múltipla, Neuromielite Óptica, Glicoproteína Mielina-Oligodendrócito, Diagnóstico

## Abstract

**Background:**

Acquired demyelinating disorders lead to overlapping visual, pyramidal, sensory, autonomic, and cerebellar deficits and may lead to severe disability. Early diagnosis and start of treatment are fundamental towards preventing further attacks and halting disability.

**Objective:**

In this paper we provide an updated overview of the differential diagnoses of acquired demyelinating disorders.

**Methods:**

We performed a critical targeted review of the diagnoses of the most prevalent demyelinating disorders: multiple sclerosis (MS), neuromyelitis optica spectrum disorders (NMOSD) and myelin oligodendrocyte glycoprotein antibody disease (MOGAD).

**Results:**

We discuss the workup, diagnostic criteria and new biomarkers currently being used for the diagnosis of these disease entities taking into account the particularities of the Brazilian population and healthcare system.

**Conclusion:**

A comprehensive analysis of medical history, physical examination, biomedical and imaging data should be performed to obtain differential diagnosis. Diagnostic criteria should be mindfully employed considering ethnic and environmental particularities of each patient.

## RESUMO

**Antecedentes:**

Doenças desmielinizantes adquiridas levam a déficits visuais, piramidais, sensitivos, autonômicos e cerebelares que se sobrepõem e podem conduzir a grave incapacidade. O diagnóstico e o início de tratamento precoces são fundamentais para a prevenção de surtos e ocorrência de incapacidade.

**Objetivo:**

Neste artigo, apresentamos uma visão geral atualizada sobre o diagnóstico diferencial de doenças desmielinizantes adquiridas.

**Métodos:**

Realizamos uma revisão crítica sobre o diagnóstico das doenças desmielinizantes mais prevalentes: esclerose múltipla (EM), doença do espectro neuromielite óptica (NMOSD) e doença associada ao anticorpo contra a glicoproteína da mielina do oligodendrócito (MOGAD).

**Resultados:**

Discutimos a investigação, os critérios diagnósticos e os novos biomarcadores atualmente empregados para o diagnóstico dessas doenças, levando em conta as particularidades da população e sistema de saúde brasileiros.

**Conclusão:**

Uma análise minuciosa do histórico médico, exame neurológico e exames biomédicos e de imagem deve ser realizada para se fazer um diagnóstico diferencial de doença desmielinizante. Critérios diagnósticos devem ser empregados cautelosamente considerando-se particularidades étnicas e ambientais de cada paciente.

## INTRODUCTION

Acquired demyelinating disorders, such as multiple sclerosis (MS), neuromyelitis optica spectrum disorders (NMOSD) and myelin oligodendrocyte glycoprotein antibody disease (MOGAD) compromise the optic nerves, brain and spinal cord and lead to a range of clinical symptoms including visual, pyramidal, sensory, autonomic, and cerebellar deficits. The diseases affect mainly young individuals and may lead to severe disability. In fact, MS is the second leading cause of disability of young adults in developed countries^1^.

Prompt diagnosis and initiation of treatment are essential towards preventing attacks and halting the accumulation of disabilities. The ability to perform accurate differential diagnoses is crucial to the good prognosis of patients, however it can be extremely challenging, as the field is dynamic and expanding, different disease entities clinically overlap and access to specific assays is still limited in certain settings.

This paper aims to provide an updated practical approach on how to perform the differential diagnoses of suspected acquired demyelinating syndromes taking into account the particularities of the Brazilian population and healthcare system.

## METHODS

We performed a targeted literature review pertaining to the diagnosis of MS, NMOSD and MOGAD. Resulting evidence was jointly critically appraised by a junior and a senior neuroimmunologist.

### Epidemiological/demographic update: prevalence of ADS in Brazil

Estimating the prevalence of demyelinating disorders in Brazil is challenging due to the absence of representative country-wide prevalence studies, heterogeneous access to qualified health systems and the ethnically diverse population. Nonetheless, a metanalysis described a national MS prevalence of 8.69/100,000 (95% CI: 6.0-12.6), with an association between the prevalence of MS and latitude of study location (OR=1.09; 95% CI: 1.04-1.14), ethnic composition (OR=1.03; 95% CI: 1.01-1.05) and weather. The authors identified a 9% increase in the prevalence rate of MS for the increase of each degree in latitude and a 3% raise in the prevalence of MS for every degree increase in the proportion of white people^2^. 

The prevalence of NMOSD is also heterogeneous and influenced by the population’s ethnical background. Different studies report prevalences in Brazil that range from 0.37 to 4.52/100,000 inhabitants with a north-south gradient decrease in the risk of developing NMOSD^3^ ^,^ ^4^. Prevalence and disease phenotypes are influenced by ethnicity, and worse clinical outcomes are associated with Asian, African and Latin American ancestry^5^. 

MOGAD incidence rates worldwide range from 0.16 to 1.4 per 100,000^6^. The proportion of MOG-IgG related acquired demyelinating syndromes (ADS) decreases with age. Higher disease incidences have been reported in pediatric cohorts (39%), than among mixed cohorts of children and adults (29%) or adults (23%)^6^. Ethnicity initially seemed to not be as important with 78-90% (MOGAD) versus 60-63% (NMOSD) of Caucasians, however Brazilian data suggests that it may play a role in the prevalence of the disease^7^ ^,^ ^8^. The nationwide estimations of the prevalence of MOGAD have not yet been published. 

### Diagnosis of demyelinating disorders

Demyelinating disorders lead to overlapping clinical syndromes. A methodical diagnostic approach, including thorough investigation of the medical history, neurological examination and complementary tests is helpful towards performing differential diagnoses (Table 1). Unfortunately, not all tests are available in the Brazilian public health system, therefore mindful investigation is suggested to prevent unnecessary financial expenses. 

**Table 1.** Diagnostic workup for demyelinating disorders.

Medical history
	Personal history of autoimmunity
	Family history of autoimmunity
	Presence of infectious or vaccinal triggers.
History of previous neurological symptoms.
Biomedical workup	*Blood*	Complete blood count, renal function, liver function, thyroid function
		Serologies (EBV, VZV, CMV, HIV, HTLV I/II, HBC, HCV, Syphillis)
		Rheumatologic profile (ANA, anti-Ro, anti-La, anti-DNA)
		AQP4-IgG
		Anti-MOG IgG
		Metabolic profile (Vitamin B12, Vitamin D, Folic acid)
	*CSF*	Total and differencial cell count
		Biochemical analysis (glucose, protein, lactate)
		Oligoclonal band profile
Image	Brain MRI	
	Spinal cord MRI	
Complementary tests	OCT	
	VEP	
	MEP/SEP	

AQP4-IgG: Anti aquaporin-4 antibody; MOG-IgG: Myelin Oligodendrocyte glycoprotein antibody.

The diagnoses can be obtained through the interpretation of the medical history, neurological examination, biomedical/imaging tests and application of current diagnostic criteria^9^,^10^ (Tables 2, 3 and 4).

**Table 2.** Diagnostic criteria for relapsing remitting multiple sclerosis.

The 2017 McDonald criteria for diagnosis of multiple sclerosis in patients with an attack at onset^9^		
Number of attacks	Number of lesions with objective clinical evidence	Additional data needed for a diagnosis of multiple sclerosis
≥2 clinical attacks	≥2	None
≥2 clinical attacks	1 (as well as clear-cut historical evidence of a previous attack involving a lesion in a distinct anatomical location	None
≥2 clinical attacks	1	Dissemination in space demonstrated by an additional clinical attack implicating a different CNS site or by MRI
1 clinical attack	≥2	Dissemination in time demonstrated by an additional clinical attack or by MRI OR demonstration of CSF-specific oligoclonal bands
1 clinical attack	1	Dissemination in space demonstrated by an additional clinical attack implicating a different CNS site or by MRI AND dissemination in time demonstrated by an additional clinical attack or by MRI OR demonstration of CSF-specific oligoclonal bands

**Table 3.** Diagnostic criteria for primary progressive multiple sclerosis.

2017 McDonald criteria for diagnosis of multiple sclerosis in patients with a disease course characterized by progression from onset (primary progressive multiple sclerosis)^9^
Primary progressive multiple sclerosis can be diagnosed in patients with:
1 year of disability progression (retrospectively or prospectively determined) independent of clinical relapse
Plus two of the following criteria:
One or more T2-hyperintense lesions characteristic of multiple sclerosis in one or more of the following brain regions: periventricular, cortical or juxtacortical, or infratentorial
Two or more T2-hyperintense lesions in the spinal cord
Presence of CSF-specific oligoclonal bands

**Table 4.** Diagnostic criteria for neuromyelitis optica spectrum disorders.

International consensus diagnostic criteria for neuromyelitis optica spectrum disorders^10^
*Diagnostic criteria for NMOSD with AQP4-IgG*
1. At least 1 core clinical characteristic
2. Positive test for AQP4-IgG using best available detection method (cell-based assay strongly recommended)
3. Exclusion of alternative diagnoses
*Diagnostic criteria for NMOSD without AQP4-IgG or NMOSD with unknown AQP4-IgG status*
1. At least 2 core clinical characteristics occurring as a result of one or more clinical attacks and meeting all of the following requirements:
a. At least 1 core clinical characteristic must be optic neuritis, acute myelitis with LETM, or area postrema syndrome
b. Dissemination in space (2 or more different core clinical characteristics)
c. Fulfillment of additional MRI requirements, as applicable
2. Negative tests for AQP4-IgG using best available detection method, or testing unavailable
3. Exclusion of alternative diagnoses
*Core clinical characteristics*
1. Optic neuritis
2. Acute myelitis
3. Area postrema syndrome: episode of otherwise unexplained hiccups or nausea and vomiting
4. Acute brainstem syndrome
5. Symptomatic narcolepsy or acute diencephalic clinical syndrome with NMOSD-typical diencephalic MRI lesions
6. Symptomatic cerebral syndrome with NMOSD-typical brain lesions
*Additional MRI requirements for NMOSD without AQP4-IgG and NMOSD with unknown AQP4-IgG status*
1. Acute optic neuritis: requires brain MRI showing (a) normal findings or only nonspecific white matter lesions, OR (b) optic nerve MRI with T2-hyperintense lesion or T1-weighted gadolinium- enhancing lesion extending over .1/2 optic nerve length or involving optic chiasm
2. Acute myelitis: requires associated intramedullary MRI lesion extending over 3 contiguous segments (LETM) OR 3 contiguous segments of focal spinal cord atrophy in patients with history compatible with acute myelitis
3. Area postrema syndrome: requires associated dorsal medulla/area postrema lesions
4. Acute brainstem syndrome: requires associated periependymal brainstem lesions

To this day, the diagnosis of MOGAD still relies on the identification of the MOG-IgG antibody in serum. Live cell-based assays are the established gold standard for the identification of the antibody, due to its superior sensitivity and specificity^11^. Unlike what is observed in MS and NMOSD, a portion of patients with MOGAD present with monophasic disease and therefore, might have a MOG IgG serostatus switch over time regardless of immunosuppressive treatment. The final diagnosis of MOGAD should account for the medical history and clinical phenotype of the patient in addition to their serostatus, as a percentage of patients with MS may present with low titers of MOG-IgG, while employed assays may not be adequately sensitive to detect low antibody titers and clear diagnostic criteria for the disease have not yet been defined.

### Differential diagnosis

The careful interpretation of medical history, physical examination and additional investigation allows the distinction between the various acquired demyelinating syndromes (Table 5). It is important to highlight that the criteria currently used for the diagnosis of MS and NMOSD were developed and validated in populations with ethnic and environmental backgrounds distinct from what is observed in the Brazilian population, which might compromise the sensitivity and specificity of the criteria. Brazilian neurologists ought to be mindful to identify “red-flags” for atypical demyelinating syndromes and systematically rule out differential diagnoses, including endemic infectious diseases such as HLTV I/II and schistosomiasis.

**Table 5.** Demographic, clinical, MRI and CSF features of demyelinating diseases of the central nervous system.

Demographic, clinical, MRI and CSF features of MS, AQP4-IgG positive NMOSD and MOG-IgG associated disease. *(Adapted from Hegen et al. Ther Adv Neurol Disord. 2020.)* ^6^			
Disease			
	MOGAD	AQP4-IgG positive NMOSD	MS
Epidemiology			
Brazilian prevalence (per 100,000)	Unknown	0.37- 4.52	8.69
Demographics			
Female:male ratio	1-2/1	8-9/1	3/1
Age at onset	More often in childhood than adulthood	>40 years	20-30 years
Clinical presentation			
Clinical presentation	ADEM-like (ADEM, MDEM, ADEM-optic neuritis, encephalitis) or opticospinal (optic neuritis, myelitis) or brainstem encephalitis	Optic neuritis, myelitis, area postrema syndrome, brainstem syndrome, narcolepsy or acute diencephalic syndrome, cerebral syndrome with NMOSD-typical brain lesions	Optic neuritis, myelitis, brainstem or cerebellar syndrome, cognitive dysfunction and symptoms caused by involvement of other MS-typical brain regions
Disease course	Monophasic and recurrent (recurrence often presents as optic neuritis)	More often recurrent than monophasic	Relapsing-remitting or chronic progressive
Magnetic resonance imaging			
Brain MRI	ADEM-like, atypical for MS (fluffy lesions or three lesions or fewer) or no brain lesions	Atypical for MS and/or lesions in the brainstem; or no brain lesions	Multiple focal white matter lesions, ovoid lesions adjacent to the lateral ventricles, Dawson fingers, U-fibre subcortical lesions, T1 hypointense lesions
Frequency of normal brain MRI at disease onset	Up to 50% (depending on type of manifestation; normal brain MRI often seen in optic neuritis)	Up 50%	NA
Spinal MRI	Long-segment lesions (>3 vertebral segments); typically involving thoracolumbar segment and conus; confined to grey matter (H sign); contrast-enhancement infrequent	Long-segment lesions (>3 vertebral segments); typically involving cervicothoracic segment; central cord predominance; contrast-enhancement frequent	Short-segment lesions (<3 vertebral segments); axial peripheral (dorsal/lateral column); contrast-enhancement frequent
Optic neuritis	Bilateral more often than unilateral, often anterior optic pathway, long lesion, often recurrent, severe, good recovery	Bilateral more often than unilateral, often posterior optic pathway, involvement of optic chiasma, long lesion, often recurrent, severe, often residual deficits	Unilateral more often than bilateral; short lesion, good recovery
Cerebrospinal fluid			
Pleocytosis	Common (>70% of patients)	Common (>70% of patients)	Moderate (<50% of patients)
Cytology	Mononuclear, but neutrophils can occur (in up to ~50% of samples)	Mononuclear, but neutrophils can occur (in up to ~50% of samples)	Mononuclear
OCBs	Rare (<10-20% of patients)	Rare (<10% of patients)	Common (>90% of patients)
Optical coherence tomography			
Axonal damage (assessed, e.g., by pRNFL decrease)	Moderate (per optic neuritis attack)	Severe (per optic neuritis attack)	Moderate (per optic neuritis attack)

### Role of novel biomarkers

Optical coherence tomography (OCT) is an imaging technique which uses infrared light in a similar manner to that of the ultrasound to measure different biological tissue's backscatter, getting micrometer-resolution images. When used in the retina, it allows the reconstruction of tomographic maps and quantification of axons of the retinal nerve fiber layer (RNFL) and neurons of the macular ganglion cell layer (mGCL)^12^. OCT has consistently been used to screen for subclinical optical abnormalities in patients with demyelinating disorders, however data shows associations between reduced RNFL/mGCL and neurodegeneration with correlations to types of MS, disability and cognitive impairment^12^ ^-^ ^15^. In addition, distinct OCT patterns can be used as diagnostic biomarkers aiding in the differential diagnosis of MOGAD from other demyelinating disorders^16^ ^,^ ^17^.

Neurofilaments (Nf) are structural proteins involved in the radial growth and stability of neurons. Studies have demonstrated that Nf-l has a value as a scientifically useful biomarker of disease activity and therapy effectiveness of groups of patients with inflammatory diseases of the CNS, such as multiple sclerosis and clinically isolated syndrome^18^ ^-^ ^22^. It is currently not commonly used in clinical practice, as its measure is modulated by body-mass index (BMI), age, and comorbidities which compromise the definition of fixed cutoffs and individually pathological levels of Nf-l^23^. Recent data has shown that Nf-l levels can be clinically employed to predict disease activity and disease-modifying therapy effectiveness in the real world setting on an individual level if percentiles and Nf-l Z scores are used^23^. Nonetheless, the description is new and the practice still not widespread.

Glial fibrillary acidic protein (GFAP) is a principal intermediate filament that forms the astrocyte cytoskeleton and is regarded as a biomarker of astrocyte injury^24^. Evidence describes its role as a potential diagnostic and prognostic biomarker in NMOSD, a known astrocytopathy, however its use is still currently limited to scientific purposes^25^ ^,^ ^26^.

## DISCUSSION

Acquired demyelinating disorders lead to a plethora of clinical syndromes which are common among distinct nosologies. In the past 20 years, anti-aquaporin 4 antibodies (AQP4-IgG), anti-MOG antibodies (MOG- IgG) and their associated disease entities, AQP-4 IgG NMOSD and MOGAD, were described. Since then, making a differential diagnosis between the most prevalent acquired demyelinating disorders has become more challenging, especially in places where environmental and genetic conditions are distinct to those of the settings where studies guiding diagnostic criteria were carried out.

A methodical approach to the diagnostic process can aid in achieving timely accurate diagnoses. In addition to a thorough medical history and neurological examination, biomedical and imaging data can provide crucial information to aid in identifying each disease. Although clinical phenotypes often overlap, integrated analysis of demographic, clinical, biomedical, and imaging data is particular to each disease and therefore should be interpreted together.

As the field further develops, new technologies and biomarkers are systematically being studied and translated from the bench to the bedside. For now, it is suggested that neurologists examine the validity, specificity, and sensitivity for individual use of each new diagnostic tool before applying it in their routine diagnostic practice. 

In conclusion, a comprehensive analysis of the diagnostic workup should be performed to obtain a differential diagnosis of an acquired demyelinating disorder. Diagnostic criteria should be mindfully employed considering ethnic and environmental particularities of each patient.
